# Short sequence motifs, overrepresented in mammalian conserved non-coding sequences

**DOI:** 10.1186/1471-2164-8-378

**Published:** 2007-10-18

**Authors:** Simon Minovitsky, Philip Stegmaier, Alexander Kel, Alexey S Kondrashov, Inna Dubchak

**Affiliations:** 1 Genomics Division, Lawrence Berkeley National Laboratory, Berkeley, CA 94720, USA; 2 BIOBASE GmbH, Halchtersche Strasse 33, D-38304 Wolfenbuettel, Germany; 3 Life Sciences Institute and Department of Ecology and Evolutionary Biology, University of Michigan, Ann Arbor, MI 48103, USA; 4 DOE Joint Genome Institute, Walnut Creek, CA 94598, USA

## Abstract

**Background:**

A substantial fraction of non-coding DNA sequences of multicellular eukaryotes is under selective constraint. In particular, ~5% of the human genome consists of conserved non-coding sequences (CNSs). CNSs differ from other genomic sequences in their nucleotide composition and must play important functional roles, which mostly remain obscure.

**Results:**

We investigated relative abundances of short sequence motifs in all human CNSs present in the human/mouse whole-genome alignments *vs*. three background sets of sequences: (i) weakly conserved or unconserved non-coding sequences (non-CNSs); (ii) near-promoter sequences (located between nucleotides -500 and -1500, relative to a start of transcription); and (iii) random sequences with the same nucleotide composition as that of CNSs. When compared to non-CNSs and near-promoter sequences, CNSs possess an excess of AT-rich motifs, often containing runs of identical nucleotides. In contrast, when compared to random sequences, CNSs contain an excess of GC-rich motifs which, however, lack CpG dinucleotides. Thus, abundance of short sequence motifs in human CNSs, taken as a whole, is mostly determined by their overall compositional properties and not by overrepresentation of any specific short motifs. These properties are: (i) high AT-content of CNSs, (ii) a tendency, probably due to context-dependent mutation, of A's and T's to clump, (iii) presence of short GC-rich regions, and (iv) avoidance of CpG contexts, due to their hypermutability. Only a small number of short motifs, overrepresented in all human CNSs are similar to binding sites of transcription factors from the FOX family.

**Conclusion:**

Human CNSs as a whole appear to be too broad a class of sequences to possess strong footprints of any short sequence-specific functions. Such footprints should be studied at the level of functional subclasses of CNSs, such as those which flank genes with a particular pattern of expression. Overall properties of CNSs are affected by patterns in mutation, suggesting that selection which causes their conservation is not always very strong.

## Background

Genomes of multicellular eukaryotes mostly consist of DNA segments which do not encode proteins. Still, a sizeable fraction of such non-coding DNA is subject to selective constraint and, thus, is conserved between species. Typically, a long intergenic region consists of alternating segments with high and low rates of evolution [1]. A variety of terms have been used to refer to slowly-evolving segments [2,3], here we will call them CNSs (conserved non-coding sequences).

A majority of mutations in segments which evolve at high rates are presumably selectively neutral or nearly-neutral. In contrast, a large fraction of mutations within CNSs must be deleterious enough to be removed by negative selection. Indeed, data on within-population genetic variability indicate that slow evolution of CNSs is due to negative selection, and not to locally reduced mutation rate [4]. In multicellular eukaryotes with compact genomes, such as *Drosophila melanogaster*, a majority of mutations affecting non-coding sequences may be removed by selection [5,6]. For large-genome organisms, such as mammals, the fraction of selectively constrained non-coding sequences is probably between 3% [7] and ~10% [8].

Obviously, CNSs must perform important biological functions, but the whole range and nature of these functions remains unknown [9]. Still, many CNSs are certainly involved in regulation of transcription, and harbor binding sites of a variety of transcription factors [10]. Thus, we can expect some short sequence motifs to be overrepresented in at least some kinds of CNSs, as this is the case for proximal promoters [11]. Indeed, analyses of samples from human CNSs demonstrated overrepresentation of some short sequence motifs [12,13].

New, powerful methods of detecting overrepresented motifs [*e. g*., [14,15]], make it possible to undertake the analysis of small-scale composition of mammalian CNSs at the genomic level. Such analysis has a potential to reveal short sequence-specific function(s) common for all human CNSs. Here, we report the results of application of discriminating matrix enumerator (DME) [14] to all strong human CNSs.

## Results

**Figure 1:**
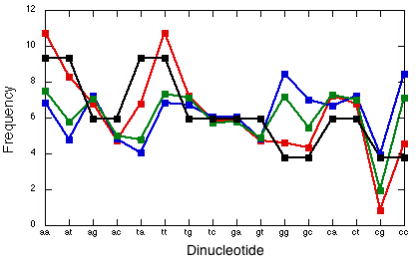
Percentages of dinucleotide frequencies, in CNSs (red), non-CNSs (green), near-promoters (lue), and random sequences (black).

We studied representation of short sequence motifs in all human CNSs against three backgrounds: unconserved or only weakly conserved segments of intergenic regions (non-CNSs), near-promoter non-coding sequences, and randomized sequences with the same nucleotide composition as that of CNSs. CNSs are relatively AT-rich [9]: frequencies of nucleotides A, T, G, and C are 30.7%, 30.7%, 19.3%, and 19.3% in CNSs, 26.3%, 26.4%, 23.6%, and 23.7% in non-CNSs, and 23.7%, 23.7%, 26.3%, and 26.3% in near-promoter sequences. Dinucleotide compositions of sequences of different classes were also substantially different (Fig. 1).

CNSs from human chromosomes with odd and even numbers were analyzed separately, to check the results for consistency. The overall lengths of CNSs were 27,112,333 on odd chromosomes and 24,962,379 on even chromosomes. Tables 1, 2, and 3 list top 30 motifs, overrepresented within CNSs over these three backgrounds. Overrepresentation was calculated as the ratio of the number of occurrences of a motif within CNSs, normalized to their overall length, over normalized number of occurrences of the motif within the background sequences.

**Table 1:** Motifs overrepresented in CNSs over non-CNSs

Odd Chromosomes			Even Chromosomes		
Motif	Number of occurrences	Overrepre-sentation	Motif	Number of occurrences	Overrepresen-tation

SYTAATTA	10620	3.45	TTAATTAV	12637	3.72
CTRATTAS	6152	3.14	TAATTRCW	12019	3.43
WGYAATTA	12596	3.09	GYAATTAS	6142	3.39
TTAATTAV	13141	3.08	TTTAATBA	15060	3.14
STAATTGV	8267	2.89	ATTAATBA	10910	3.07
VWGCTAAT	10503	2.84	TAATTWGM	10885	3.04
TTTAATBA	15800	2.77	GMWTAATT	9941	2.97
GMWTAATT	10290	2.72	CWTAATKA	10028	2.94
TAATTATV	10100	2.72	ATTAAWTT	11570	2.85
STTAATKG	5905	2.71	TTAATBAT	10115	2.79
ATTVAATT	12177	2.68	CWKTAATT	13079	2.75
ATTAATBA	11006	2.61	VWGCTAAT	9823	2.71
CWKTAATT	13577	2.59	CMATWAAT	10129	2.65
ATAATTAV	10536	2.58	ATTTVATT	15715	2.64
SMAATTAA	12754	2.57	CAATTRCH	8188	2.61
SBTAATGA	8828	2.56	MCWAATTA	9605	2.61
VATTWGCA	14265	2.53	ATTWWGCA	9959	2.61
TWAATCAR	10639	2.52	GKTAATTW	9019	2.59
AATTAVTT	12668	2.51	AATTAMCW	10053	2.58
GTAATTMM	7484	2.49	MATTDGCA	13694	2.58

**Table 2:** Motifs overrepresented in CNSs over near-promoter sequences

Odd Chromosomes			Even Chromosomes		
Motif	Number of occurrences	Overrepre- sentation	Motif	Number of occurrences	Overrepresen-tation

STAATTAS	7576	4.55	SYTAATTA	9852	4.26
TTAATKAR	17516	4.33	TTAATTAD	14561	4.07
GBTAATKA	12299	3.96	CTRATTAS	5744	3.90
VTAATTGM	10174	3.91	ATTAATGN	9762	3.74
TTTMATKA	19449	3.86	TAATTATD	11760	3.73
MTTMATTA	13688	3.82	TTTAATDA	16633	3.66
AATKYAAT	15204	3.73	ATAATTAB	9233	3.62
TTAATKGV	12925	3.72	TAATKSAA	10418	3.59
RTAATKAA	13613	3.68	STAATTGV	7823	3.55
MMTAATTA	12518	3.68	GYAATWAA	10608	3.55
TSTAATTW	14964	3.49	TGYAATTW	13322	3.51
AATKMATT	18824	3.48	AATGMWTT	15412	3.49
TGATWAAW	12898	3.46	AGYAATTW	12585	3.41
KATAATKA	10739	3.46	AATTDATT	14693	3.39
CATTAAKV	10838	3.42	AATTATAD	10379	3.36
CATWAWTT	14599	3.39	TWAATTGR	8896	3.35
CATTWAAW	19325	3.37	AWTARCAT	9601	3.35
CAATTAKV	9515	3.33	TAATTHAT	12789	3.34
ATRATTYA	13356	3.30	CWTTAATR	9114	3.32
ATTTYMAT	20983	3.29	ATTSMATT	11547	3.27

**Table 3:** Motifs overrepresented in CNSs over randomized sequences

Odd Chromosomes			Even Chromosomes		
Motif	Number of occurrences	Overrepre-sentation	Motif	Number of occurrences	Overrepre-sentation

CWGSCWGS	32472	7.50	CWGSCWGV	38927	5.78
SCCHGSCH	42207	5.68	SCCWGGSN	33122	5.63
GGSWGGSN	39555	5.55	CYCWSCCH	33976	5.50
CWGSCCWS	24103	5.52	RGCWGSCH	30738	4.95
RGTCCTBY	22100	5.45	GGSDGRGV	34873	4.93
GRGSWGRG	25293	5.36	CWGSCYCH	29902	4.78
CCYYYCCH	40727	5.22	CWSCWGGV	31840	4.73
SCCWGGRV	33839	5.20	SCWGCWGV	30968	4.71
CWGSCYCH	36409	5.04	CWGGGRRV	31866	4.64
SCWGGGSN	36038	5.03	CWGRGSCH	28886	4.61
SCHGSCCH	36013	4.91	CCWGGRRV	31578	4.61
CWGRGSCH	35318	4.77	SCHGGSCH	28689	4.50
SCYCWGCH	34141	4.56	GGRARGRR	29240	4.47
NCAGCTGN	32928	4.52	RRGGCWGV	30772	4.44
CAGCTGNN	32867	4.51	RGGGRARR	29828	4.41
TWACWGAA	14781	4.48	GVWGGGRR	31019	4.37
RGGGRRAR	32929	4.42	CYCYVSCC	19097	4.37
CWGSAGSY	24140	4.37	KCCWSCCH	26417	4.33
SCWGGRAR	32065	4.37	CAGCYSNG	16617	4.28
GGARRGRR	33390	4.37	KKGGCWGV	28051	4.13

In order to study a possible similarity of the overrepresented CNS motifs with known binding sites for transcription factors (TF), we applied our recently developed method m2transfac [16], and compared all the motifs found at the previous step with the TRANSFAC library of positional weight matrices (PWMs). Relatively few matches between the motifs and the TF matrices were found. Out of 12000 motifs reported at the previous step as being overrepresented in CNS versus the three different backgrounds, we have identified just 20 motifs that match TF matrices with E-values lower than 0.001 and satisfy factor class-specific cut-offs (Table 4). The majority of these matches involved matrices for the factors of "Forkhead DNA-binding domain", especially of the FOX family, which were repeatedly found over two rather different backgrounds: of non-CNSs and randomized sequences. Among the motifs found over the background of near-promoter sequences, there was only one that matched a PWM.

**Table 4:** Motifs found matching transcription factor PWMs from TRANSFAC

**Accession**	**Consensus/ID**	**Factor class**	**Taxon**	**Binding factors**
**acns even**				
DME280	ATAAACAN	Forkhead DNA-binding domain	Vertebrate	FOXI1a,FOXF1,FOXL1,FOXO4
DME424	WGTAAAYA	Forkhead DNA-binding domain	Vertebrate	FOXC1,FOXA4a,HNF-3beta
DME768	WTGTCATV	Basic region + leucine zipper (bZIP)	Nematode	Skn-1
DME1427	WGTCATSM	Basic region + leucine zipper (bZIP)	Nematode	Skn-1
**acns odd**				
DME27	VATTWGCA	POU	Vertebrate	POU2F1
DME349	ATAAACAN	Forkhead DNA-binding domain	Vertebrate	FOXI1a,FOXF1,FOXL1,FOXO4
DME1014	GTMAACAD	Forkhead DNA-binding domain	Vertebrate	FOXD1,HNF-3beta,FOXO1a
DME1700	CCAATMAB	DNA-binding domain with Histone fold	Fungal	HAP2,HAP3,HAP4
**promoters even**				
**promoters odd**				
DME1268	STGASTYA	Basic region + leucine zipper (bZIP)	Vertebrate	NF-E2,AP-1
**random even**				
DME90	VCAGATGN	Basic region + helix-loop-helix motif	Vertebrate	ITF-2,Tal-1beta
DME94	CATCTGBN	Basic region + helix-loop-helix motif	Vertebrate	ITF-2,Tal-1beta,E47
DME765	RTGWSTCA	Basic region + leucine zipper (bZIP)	Vertebrate	NF-E2,AP-1,Fos,Jun,Fra
DME1106	TGTTBACW	Forkhead DNA-binding domain	Vertebrate	HNF-3beta
DME1111	ATAAACAH	Forkhead DNA-binding domain	Vertebrate	FOXI1a,FOXF1,FOXL1,FOXO4
DME1920	CCACGTGG	Basic region + helix-loop-helix motif	Plant, Vertebrate	PIF3,c-Myc:Max
**random odd**				
DME11	CAGCTGNN	Basic region + helix-loop-helix motif	Vertebrate	AP-4
DME456	MAYAAACA	Forkhead DNA-binding domain	Vertebrate	FOXF1
DME790	TATGVAAA	POU	Vertebrate	POU2F1
DME930	ATAAAYAT	Forkhead DNA-binding domain	Vertebrate, Insect	FOXI1a,Croc
DME1145	TGTTBACW	Forkhead DNA-binding domain	Vertebrate	HNF-3beta

## Discussion

We treated all human CNSs as a single class of sequences. Comparison of this class against three different backgrounds demonstrates that many short sequence motifs are substantially overrepresented within CNSs (Tables 1, 2, 3). CNSs from odd- and from even-numbered human chromosomes show very similar patterns, which is consistent with the lack of any large-scale heterogeneity within CNSs. At a first glance, these results may seem to suggest that CNSs as a whole possess some complex sequence pattern(s), with possible implications for their functioning. However, this is probably not the case. Instead, the results can be explained by simple, generic properties of CNSs.

Indeed, when CNSs are analyzed against a background of non-CNSs (Table 1) or of near-promoter sequences (Table 2), almost all overrepresented motifs possess two common features: (i) they are AT-rich (consist of 75% or more of A and/or T) and (ii) they contain runs of A's and/or T's. Feature (i) simply reflects a well-known, although poorly understood, fact that CNSs are more AT-rich than the genome as a whole [9,17] or that these two classes of background sequences. Feature (ii) appears to be due to general excess of AA and TT dinucleotides in CNSs, relatively to corresponding random sequences. This tendency of A's and T'e to clump is probably due to patterns in mutation, and not to any functional constraint. Indeed, context-dependence of spontaneous mutation in mammals tends to produce runs of A's and T's, because at a site preceded and followed by A's (T's) T>A (A>T) transversions are ~2 times more common than A>T transversions [18,19]; Table 2.

Obviously, it is neccessary to consider CNSs against a background of the same nucleotide composition, as otherwise the impact of different compositions is the leading factor causing overrepresentation of some motifs. When CNSs are analyzed against a background of random sequences of the same, AT-rich, nucleotide composition, the results are very different (Table 3), and overrepresented motifs can be naturally subdivided into two classes. The first, larger class contains a variety of GC-rich motifs which, however, are devoid of CpG dinucleotides and are correspondingly enriched with CpA and CpT dinucleotides and with CWG short motif. The second, smaller class contains several motifs which are either purine- or pyrimidine-rich. Overrepresentation of motifs from the first class appear to be due to two simple factors: i) the presence, within CNSs, of short GC-rich segments and ii) hypermutability of CpG dinucleotides [18]. Indeed, CNSs are depleted of CpG's more than the other two classes of genomic sequences (Fig. 1), which might reflect strong methilation of CNSs. Overrepresentation of motifs of the second class simply reflects a well-known [20], although poorly understood, abundance of short segments with strong purine/pyrimidine imbalance between the two DNA stands within the human genome.

The analysis of all human CNSs does not reveal clear "global" patterns consistent with overrepresentation of specific, functional motifs. A small number of the observed overrepresented motifs are similar to Position Weight Matrices (PWMs) from TRANSFAC database [21] (Table 4). Among them, the strongest similarity was to the PWMs of FOX and POU families of factors which are characterized by a specific AT-rich pattern. In order to test if the identification of FOX-domain matrices is merely an effect of the general AT-richness of the CNS regions we check carefully results of alignments of all other "AT-rich" matrices in TRANSFAC. There are approximately 64% of matrices in TRANSFAC with overall AT composition higher then 50%. 16 of them are characterised by the same and even higher AT-composition then any of the FOX and POU-domain matrices (e.g. matrices for such factors as TBP, Lhx3, Evi-1, Nkx3-1 and others). Nevertheless, non of them gave statistically significant results of the alignments with the motifs under study. This confirms the similarity of some motifs from the list specifically to the FOX- and POU-domain matrices. The FOX factors are involved in many cellular processes and often control very first steps of organism development as well as cell cycle and differentiation; e. g. FOXF1 is highly expressed in mouse embryonic extraembryonic and lateral mesoderm [22] and control murine gut development [23]; FOXD1 is predominantly expressed in embryonic forebrain neuroepithelium, head mesenchyme and adrenal cortex [24] and controls normal brain and kidney morphogenesis and cellularity in the renal capsule [25]; FOXO1 governs cell growth in the heart [26]. Factors of other families, such as POU and bZIP are often involved in regulation of basic cell cycle machinery; e.g. POU2F1 is an ubiquitous factor involved in stimulation of replication [27] and also participates in early mouse embryogenesis [28]. In summary, it might be tempting to speculate that at least some motifs overrepresented in all CNSs may play crucial role in organizing the process of development of the vertebrate organisms. However, the number of such motifs is not high., More specific classes of CNSs, such as those adjacent to genes with a particular pattern in expression [11,12] should be considered in order to find a larger number of functional motifs.

In contrast, small-scale composition of human CNSs, considered as a whole, is strongly affected by patterns in mutation – hypermutability of CpG's and the tendency for A's and T's to form runs. This is unexpected because CNSs must be under negative selection which can overcome any impact of mutation [4]. Apparently, selective constraint on the evolution of individual nucleoitide site can be quite weak even within strongly conserved CNSs.

## Conclusion

Abundance of short sequence motifs in all human CNSs is mostly dictated by their general features: overall AT-richness of CNSs, runs of A's and T's, GC-rich regions, avoidance of CpG's, and local purine/pyrimidine imbalance of the DNA strands. Apparently, CNSs as a whole are too broad a class to display strong overrepresentation of specific motifs. Instead, such motifs must be sought within subclasses of CNSs. In particular, tissue-specificity of expression of the genes adjacent to a CNS must be taken into account.

## Methods

We used the VISTA pipeline infrastructure [29] with Shuffle-LAGAN glocal chaining algorithm [30] applied to local alignments produced by translated BLAT [31] for the construction of genome-wide pairwise human/mouse alignment. The level of conservation in the alignment was evaluated with the computational algorithm Gumby [32] that makes minimal assumptions about the statistical features of conserved noncoding regions and treating the sequence alignment as its own training set. Gumby [32] proceeds through five steps:

1. Noncoding regions in the input alignment are used to estimate the neutral mismatch frequency *p*N between each pair of aligned sequences. This is done simply by counting the number of mismatches in nonexonic positions and dividing by the number of aligned nonexonic positions.

2. A log-odds scoring scheme for constrained versus neutral evolution is then independently initialized for the pair of sequences, based on the assumption that the mismatch frequency *p*C in constrained regions equals *p*N/*R*, where the ratio *R *is an arbitrary parameter. For example, if *R *= 3/2 (default value), constrained regions are expected to evolve at 2/3 times the neutral rate, until sequence divergence begins to saturate.

The log-odds mismatch score for the sequence pair is then given by S0 = log((*p*N/*R*)/*p*N) = -log(*R*), and the match score is S1 = log((1 - *p*N/*R*)/(1 - *p*N)). The default *R*-ratio (1.5) was selected to optimize the sensitivity-specificity tradeoff in detecting empirically defined regulatory elements in the *SCL *locus. Gap characters in the alignment are assigned a weighted average of mismatch and match scores: SG = *p*NS0 + (1 - *p*N)S1.

3. Each alignment column is scored as a sum of pairwise log-odds scores. The resulting conservation score fulfills the requirements of Karlin-Altschul statistics, in that positive column scores are possible, though the average column score is negative [33].

4. Conserved regions appear as stretches of alignment columns with a high aggregate score.

5. The aggregate score of the alignment columns in each conserved region is translated into a *P*-value using Karlin-Altschul statistics. As is the case with the BLAST algorithm [34], the *P*-value of a given conserved element varies with the size of the search space, since one is more likely to find a given degree of conservation by random chance in a long alignment than in a short alignment. To make the *P*-values comparable across alignments of different lengths, Gumby normalizes them to refer to a fictitious fixed-length alignment with the same statistical properties as the true alignment. The 10-kb *P*-value is related to the expected number of false positives in a 10-kb region (i.e. the 10-kb *E*-value) as follows: *P *= 1-exp(-*E*). When *P *<< 1, *P *≈ *E*. Thus, the *P*-value also doubles as an estimate of the false-positive rate.

Intervals with P-value threshold of 0.01 produced a set of 144,165 highly conserved sequences that totaled 49 Mb in length. We eliminated all conserved regions that coincide with the coding evidence provided by the UCSC data sets of mRNA, human spliced EST and human EST. We excluded CNSs located within (-1000, +1000) from the start and end of transcription.

Non-CNSs were defined as regions that have human/mouse alignment, conserved below 50% in a 100 bp window and not containing repeats and coding evidences. Random sequences were generated using standard C library pseudo-random generator. Overrepresentation of motifs in different random sequences was calculated using DME [14] (see Additional File 1). DME identifies motifs, represented as position weight matrices that are overrepresented in one set of sequences relative to another set. The ability to directly optimize relative overrepresentation is a unique feature of DME, making DME an ideal tool for comparing two sets. In all of studies we compared 8-mers (parameter w = 8) and bits/column bound was set to 1.6 (parameter i = 1.6).

DME motifs were compared to the TRANSFAC^® ^database with the m2transfac program [16]. The program retrieves all non-overlapping pairwise ungapped alignments of a query matrix and a TRANSFAC matrix satisfying a given threshold. The primary similarity measure is an alignment score which combines Kullback-Leibler divergence with a scoring system that was previously applied successfully to comparison of Hidden Markov Models [35]

*S*(*p*, *q*) = *C*(*p*, *q*) - *D*(*p*, *q*)

with

C(p,q)=log⁡2∑i=14piqiri
 MathType@MTEF@5@5@+=feaafiart1ev1aaatCvAUfKttLearuWrP9MDH5MBPbIqV92AaeXatLxBI9gBaebbnrfifHhDYfgasaacH8akY=wiFfYdH8Gipec8Eeeu0xXdbba9frFj0=OqFfea0dXdd9vqai=hGuQ8kuc9pgc9s8qqaq=dirpe0xb9q8qiLsFr0=vr0=vr0dc8meaabaqaciaacaGaaeqabaqabeGadaaakeaacqWGdbWqcqGGOaakcqWGWbaCcqGGSaalcqWGXbqCcqGGPaqkcqGH9aqpcyGGSbaBcqGGVbWBcqGGNbWzdaWgaaWcbaGaeGOmaidabeaakmaaqahabaWaaSaaaeaacqWGWbaCdaWgaaWcbaGaemyAaKgabeaakiabdghaXnaaBaaaleaacqWGPbqAaeqaaaGcbaGaemOCai3aaSbaaSqaaiabdMgaPbqabaaaaaqaaiabdMgaPjabg2da9iabigdaXaqaaiabisda0aqdcqGHris5aaaa@48E5@

D(p,q)=12(∑i=14pilog⁡piqi+∑i=14qilog⁡qipi)
 MathType@MTEF@5@5@+=feaafiart1ev1aaatCvAUfKttLearuWrP9MDH5MBPbIqV92AaeXatLxBI9gBaebbnrfifHhDYfgasaacH8akY=wiFfYdH8Gipec8Eeeu0xXdbba9frFj0=OqFfea0dXdd9vqai=hGuQ8kuc9pgc9s8qqaq=dirpe0xb9q8qiLsFr0=vr0=vr0dc8meaabaqaciaacaGaaeqabaqabeGadaaakeaacqWGebarcqGGOaakcqWGWbaCcqGGSaalcqWGXbqCcqGGPaqkcqGH9aqpdaWcaaqaaiabigdaXaqaaiabikdaYaaadaqadaqaamaaqahabaGaemiCaa3aaSbaaSqaaiabdMgaPbqabaGccyGGSbaBcqGGVbWBcqGGNbWzdaWcaaqaaiabdchaWnaaBaaaleaacqWGPbqAaeqaaaGcbaGaemyCae3aaSbaaSqaaiabdMgaPbqabaaaaOGaey4kaSYaaabCaeaacqWGXbqCdaWgaaWcbaGaemyAaKgabeaakiGbcYgaSjabc+gaVjabcEgaNnaalaaabaGaemyCae3aaSbaaSqaaiabdMgaPbqabaaakeaacqWGWbaCdaWgaaWcbaGaemyAaKgabeaaaaaabaGaemyAaKMaeyypa0JaeGymaedabaGaeGinaqdaniabggHiLdaaleaacqWGPbqAcqGH9aqpcqaIXaqmaeaacqaI0aana0GaeyyeIuoaaOGaayjkaiaawMcaaaaa@5FCB@

In equation (2), r is background model which is set to the uniform distribution. Equation (2) is based on the column score derived in [33]. The term assigns a positive score to similar distributions and tends towards zero for less conserved positions. Equation (3) is a symmetrized relative entropy or Kullback-Leibler (KL) divergence. Relative entropy was used previously in applications for classification of protein as well as nucleotide patterns [36,37]. The m2transfac scoring system combines the advantages of both measures. The KL divergence directly assesses the difference of two distributions and therefore increases specificity for similar distributions, but makes no distinction on the basis of their conservation, which is however a property of the column score.

The m2transfac output provides E-values, the number of alignments with greater or equal score expected from searching a database with 1000 matrices. These are derived for each TRANSFAC PWM from score distribution estimates based on large-scale searches of a random matrix library. Furthermore, we apply the transcription factor classification that was developed in our group [38,39] to gather matrices according to DNA-binding domain classes of their binding factors and derive factor class-specific score thresholds. We define 57 matrix groups, 15 of which comprise matrices which cannot be associated with a particular factor class, e.g. the barbiturate-inducible element, or whose binding factors are so far not assigned to a protein-structural class. Some matrices occur in more than one class if TFs of different classes are annotated as binding factors, or binding factors possess multiple DNA-binding domain types. For each PWM, score thresholds are defined at three levels of stringency above the score of the first observed false positive in a search of the TRANSFAC database.

## Authors' contributions

SM designed and carried out the computational experiments; PS developed the program and analyzed TRANSFAC PWMs, A Kel provided biological insight and actively participated in discussion of the project and writing the paper, A Kondrashov and ID designed and led the project. All authors have read and approved the final version of the manuscript.

## Supplementary Material

Additional file 1 Overrepresented motifs when two random sets are compared. The data provided represent comparison of two randomized sets of sequences. Click here for file

## References

[B1] Shabalina SA, Kondrashov AS (1999). Pattern of selective constraint in C. elegans and C. briggsae genomes. Genet Res.

[B2] Dermitzakis ET, Reymond A, Scamuffa N, Ucla C, Kirkness E, Rossier C, Antonarakis SE (2003). Evolutionary discrimination of mammalian conserved non-genic sequences (CNGs). Science.

[B3] Margulies EH, Blanchette M, Haussler D, Green ED (2003). Identification and characterization of multi-species conserved sequences. Genome Res.

[B4] Drake JA, Bird C, Nemesh J, Thomas DJ, Newton-Cheh C, Reymond A, Excoffier L, Attar H, Antonarakis SE, Dermitzakis ET (2006). Conserved noncoding sequences are selectively constrained and not mutation cold spots. Nat Genet.

[B5] Halligan DL, Keightley PD (2006). Ubiquitous selective constraints in the Drosophila genome revealed by a genome-wide interspecies comparison. Genome Res.

[B6] Andolfatto P (2005). Adaptive evolution of non-coding DNA in Drosophila. Nature.

[B7] Waterston RH, Lindblad-Toh K, Birney E, Rogers J, Abril JF, Agarwal P, Agarwala R, Ainscough R, Alexandersson M, An P (2002). Initial sequencing and comparative analysis of the mouse genome. Nature.

[B8] Shabalina SA, Ogurtsov AY, Kondrashov VA, Kondrashov AS (2001). Selective constraint in intergenic regions of human and mouse genomes. Trends Genet.

[B9] Dermitzakis ET, Reymond A, Antonarakis SE (2005). Conserved non-genic sequences – an unexpected feature of mammalian genomes. Nat Rev Genet.

[B10] Hardison RC (2000). Conserved noncoding sequences are reliable guides to regulatory elements. Trends Genet.

[B11] Smith AD, Sumazin P, Xuan Z, Zhang MQ (2006). DNA motifs in human and mouse proximal promoters predict tissue-specific expression. Proc Natl Acad Sci USA.

[B12] Bernat JA, Crawford GE, Ogurtsov AY, Collins FS, Ginsburg D, Kondrashov AS (2006). Distant conserved sequences flanking endothelial-specific promoters contain tissue-specific DNase-hypersensitive sites and over-represented motifs. Hum Mol Genet.

[B13] Kondrashov AS, Shabalina SA (2002). Classification of common conserved sequences in mammalian intergenic regions. Hum Mol Genet.

[B14] Smith AD, Sumazin P, Zhang MQ (2005). Identifying tissue-selective transcription factor binding sites in vertebrate promoters. Proc Natl Acad Sci USA.

[B15] Liu Y, Wei L, Batzoglou S, Brutlag DL, Liu JS, Liu XS (2004). A suite of web-based programs to search for transcriptional regulatory motifs. Nucleic Acids Res.

[B16] Stegmaier P, Kel A, Wingender E http://www.gene-regulation.com/pub/programs.html#m2transfac.

[B17] Walter K, Abnizova I, Elgar G, Gilks WR (2005). Striking nucleotide frequency pattern at the borders of highly conserved vertebrate non-coding sequences. Trends Genet.

[B18] Hwang DG, Green P (2004). Bayesian Markov chain Monte Carlo sequence analysis reveals varying neutral substitution patterns in mammalian evolution. Proc Natl Acad Sci USA.

[B19] Kondrashov FA, Ogurtsov AY, Kondrashov AS (2006). Selection in favor of nucleotides G and C diversifies evolution rates and levels of polymorphism at mammalian synonymous sites. J Theor Biol.

[B20] Lander ES, Linton LM, Birren B, Nusbaum C, Zody MC, Baldwin J, Devon K, Dewar K, Doyle M, FitzHugh W (2001). Initial sequencing and analysis of the human genome. Nature.

[B21] Matys V, Kel-Margoulis OV, Fricke E, Liebich I, Land S, Barre-Dirrie A, Reuter I, Chekmenev D, Krull M, Hornischer K (2006). TRANSFAC and its module TRANSCompel: transcriptional gene regulation in eukaryotes. Nucleic Acids Res.

[B22] Peterson RS, Lim L, Ye H, Zhou H, Overdier DG, Costa RH (1997). The winged helix transcriptional activator HFH-8 is expressed in the mesoderm of the primitive streak stage of mouse embryos and its cellular derivatives. Mech Dev.

[B23] Ormestad M, Astorga J, Landgren H, Wang T, Johansson BR, Miura N, Carlsson P (2006). Foxf1 and Foxf2 control murine gut development by limiting mesenchymal Wnt signaling and promoting extracellular matrix production. Development.

[B24] Hatini V, Huh SO, Herzlinger D, Soares VC, Lai E (1996). Essential role of stromal mesenchyme in kidney morphogenesis revealed by targeted disruption of Winged Helix transcription factor BF-2. Genes Dev.

[B25] Levinson RS, Batourina E, Choi C, Vorontchikhina M, Kitajewski J, Mendelsohn CL (2005). Foxd1-dependent signals control cellularity in the renal capsule, a structure required for normal renal development. Development.

[B26] Ni YG, Berenji K, Wang N, Oh M, Sachan N, Dey A, Cheng J, Lu G, Morris DJ, Castrillon DH (2006). Foxo transcription factors blunt cardiac hypertrophy by inhibiting calcineurin signaling. Circulation.

[B27] Schreiber E, Matthias P, Müller MM, Schaffner W (1988). Identification of a novel lymphoid specific octamer binding protein (OTF-2B) by proteolytic clipping bandshift assay (PCBA). EMBO J.

[B28] Scholer HR, Balling R, Hatzopoulos AK, Suzuki N, Gruss P (1989). Octamer binding proteins confer transcriptional activity in early mouse embryogenesis. EMBO J.

[B29] Frazer KA, Pachter L, Poliakov A, Rubin EM, Dubchak I (2004). VISTA: computational tools for comparative genomics. Nucleic Acids Res.

[B30] Brudno M, Malde S, Poliakov A, Do CB, Couronne O, Dubchak I, Batzoglou S (2003). Glocal alignment: finding rearrangements during alignment. Bioinformatics.

[B31] Kent WJ (2002). BLAT–the BLAST-like alignment tool. Genome Res.

[B32] Prabhakar S, Poulin F, Shoukry M, Afzal V, Rubin EM, Couronne O, Pennacchio LA (2006). Close sequence comparisons are sufficient to identify human cis-regulatory elements. Genome Res.

[B33] Karlin S, Altschul SF (1990). Methods for assessing the statistical significance of molecular sequence features by using general scoring schemes. Proc Natl Acad Sci USA.

[B34] Altschul SF, Gish W, Miller W, Myers EW, Lipman DJ (1990). Basic local alignment search tool. J Mol Biol.

[B35] Soding J (2005). Protein homology detection by HMM-HMM comparison. Bioinformatics.

[B36] Roepcke S, Grossmann S, Rahmann S, Vingron M (2005). T-Reg Comparator: an analysis tool for the comparison of position weight matrices. Nucleic Acids Res.

[B37] Sjölander K (1998). Phylogenetic inference in protein superfamilies: analysis of SH2 domains. Proc Int Conf Intell Syst Mol Biol.

[B38] Stegmaier P, Kel AE, Wingender E (2004). Systematic DNA-binding domain classification of transcription factors. Genome Inform.

[B39] Wingender E (1997). [Classification of eukaryotic transcription factors]. Mol Biol (Mosk).

