# Supplementary material for: Short sequence motifs, overrepresented in mammalian conserved non-coding sequences
Source: BMC Genomics. 2007 Oct 18;8:378. doi: 10.1186/1471-2164-8-378 (PMC2176071; doi:10.1186/1471-2164-8-378)
Supplement: Additional file 1 — Overrepresented motifs when two random sets are compared. The data provided represent comparison of two randomized sets of sequences. [file 1471-2164-8-378-S1.doc]

Overrepresented motifs when two random sets are compared.

| GTSGSSCN | 3754 | 1.23 |
| --- | --- | --- |
| YNSCCGGM | 4009 | 1.23 |
| CCCRASYK | 3123 | 1.22 |
| GSSCSMCV | 4287 | 1.22 |
| CSRGCTSD | 4010 | 1.21 |
| CBTGSGKM | 4412 | 1.21 |
| SDGYCSGT | 3950 | 1.21 |
| SSGCGWTV | 4088 | 1.21 |
| ACCSGKVM | 4334 | 1.2 |
| SBGRCCRA | 4392 | 1.2 |
| SGSYGSGV | 4270 | 1.2 |
| GTGSGHGN | 3914 | 1.2 |
| SCCGGADN | 4019 | 1.2 |
| GRSGCSYM | 3993 | 1.2 |
| CCACGHSN | 3982 | 1.19 |
| CMTSCSCD | 3921 | 1.19 |
| MACGBGSR | 4291 | 1.19 |
| VSCCACRM | 4340 | 1.19 |
| GGGMSKBT | 4298 | 1.19 |
| CCGGRKVW | 4336 | 1.19 |
| BMCCGGTN | 4361 | 1.19 |
| TCSRYSGA | 3988 | 1.19 |
| SGCGTKRV | 4313 | 1.19 |
| CGTGYSSD | 3918 | 1.19 |
| CGYGSAVM | 4277 | 1.19 |
| MCBCWGGR | 4284 | 1.19 |
| KGBASCGR | 4396 | 1.18 |
| SGADGSAA | 3730 | 1.18 |
| GMGCTCNV | 4362 | 1.18 |
| CGRTCCBN | 4241 | 1.18 |
| KSCGMGVA | 4290 | 1.18 |
| GSGSATSV | 4040 | 1.18 |
| CVSGCRMA | 4310 | 1.18 |
| GASSGMKT | 3746 | 1.18 |
| CTCCVSKR | 4346 | 1.18 |
| GGVCSARM | 4315 | 1.18 |
| CSGWACSV | 4111 | 1.18 |
| GCGWGKMV | 4369 | 1.18 |
| SMGGGARV | 4331 | 1.18 |
| BCSCGSAW | 4137 | 1.18 |
| CGCASSVW | 4083 | 1.18 |
| VGCYGGAN | 4340 | 1.18 |
| DMMCCGGR | 4125 | 1.18 |
| GGCWKASR | 3875 | 1.18 |
| YCRCMCGH | 4075 | 1.18 |
| CGGMNVGT | 4242 | 1.17 |
| GCAYDCGD | 4031 | 1.17 |
| GGDCKCSW | 3924 | 1.17 |
| MVCTCCCN | 4330 | 1.17 |
| STCGBGRM | 4308 | 1.17 |
